# Risk of venous and arterial thromboembolic events associated with tyrosine kinase inhibitors in advanced thyroid cancer: a meta-analysis and systematic review

**DOI:** 10.18632/oncotarget.24599

**Published:** 2018-02-26

**Authors:** Yang Bai, Jing-Yan Li, Jie Li, Bo Zhang, Yong-Hong Liu, Bu-Yong Zhang, Jian Jing

**Affiliations:** ^1^ Department of Thyroid Surgery, Cangzhou Central Hospital, Cangzhou, Hebei, China; ^2^ Department of Ultrasonography, Cangzhou Hospital of Integrated TCM-WM, Cangzhou, Hebei, China; ^3^ Department of Ultrasonography, Cangzhou Hospital of Integrated TCM-WM, Cangzhou, Hebei, China

**Keywords:** tyrosine kinase inhibitors, toxicity, arterial thromboembolic events, venous thromboembolic events, meta-analysis

## Abstract

**Aims:** To assess the incidence and risk of arterial and venous thromboembolic events (ATEs and VTEs) associated with tyrosine kinase inhibitors (TKIs) in advanced thyroid cancer patients.

**Materials and Methods:** We comprehensively searched EMBASE, Pubmed, and Cochrane Library for relevant trials. Prospective clinical trials evaluating the role of TKIs alone in advanced thyroid cancer patients were included for analysis. Data on high-grade VTEs and ATEs were extracted. The pooled incidence, Peto odds ratio (Peto OR), and 95% confidence intervals (CIs) were pooled according to the heterogeneity of included trials.

**Results:** A total of 1,781 patients from 12 trials, including four randomized controlled trials and eight phase II single arm trials, were included for analysis. Our results showed that the overall incidence of high-grade ATEs and VTEs associated with TKIs were 1.4% and 3.3%, and TKIs treatment in advanced TCs patients significantly increased the risk of developing high-grade ATEs (Peto OR 4.72, 95% CI: 1.18–18.95, *p* = 0.029), but not for high-grade VTEs (Peto OR 1.36, 95% CI: 0.51–3.64, *p* = 0.54) when compared to placebo. The most common specific causes of ATEs were myocardial infarction (28.6%) and ischemic cerebrovascular events (21.4%), respectively.

**Conclusions:** TKIs treatment in advanced thyroid cancer significantly increases the risk of developing high-grade ATEs but not for VTEs. Clinicians should be cautious about the risk of severe ATEs associated with TKIs to maximize the benefits and minimize the toxicities.

## INTRODUCTION

Thyroid cancers (TCs) are the most common endocrine malignancy [1]. Due to the improvement of diagnostic imaging, as well as emergence of environmental and genetic influences, the incidence of TCs has been rapidly rising worldwide during the past decades [1–3]. TCs could be distinguished into well-differentiated (DTCs), poorly differentiated (PDTC), and anaplastic (ATC), which represent 85%, 5%, and 3% of the entire TCs, respectively. Generally, TCs have favorable prognosis with a long-term survival rate up to 92%, following classical treatments including surgery, radioactive iodine (RAI) therapy and thyroid-stimulating hormone (TSH) suppression treatment [4]. Nevertheless, 7–23% of patients would develop distant metastases during the disease course [5], and it is of no value in patients whose disease do not concentrate iodide, and the prognosis for those patients with primary or secondary radioiodine (RAI)-refractory thyroid carcinoma becomes significantly poorer, with 10 year survival of about 10% [6, 7]. According to the American Thyroid Association (ATA) thyroid cancer guidelines [8], RAI refractory disease is defined as poor avidity of tumors on RAI scans and disease progression despite radioactive iodine uptake in the following 6 to 12 months after therapy indicate refractoriness to therapy.

Currently, the treatment of advanced RAI-refractory TCs is challenging. Until now, doxorubicin remains the single most effective and approved cytotoxic chemotherapy for the treatment of these patients, but the efficacy is limited [9, 10]. Recently, the treatment options for RAI-refractory TCs have been expanded with the advent of molecular therapies targeting the signaling pathways involved in the pathogenesis and proliferation of TCs [11–13]. To date, four orally active, small molecule, multi-targeted receptor tyrosine kinase inhibitors (TKIs), sorafenib, vandetanib, cabozantinib and lenvatinib, have been approved for the treatment of locally recurrent or metastatic progressive TCs [14]. Lenvatinib has also been approved in Japan for the treatment of unresectable thyroid cancer. In addition, several other TKIs, including sunitinib, pazopanib, and axitinib, have been investigated in RAI-refractory TCs. Although TKIs are generally well tolerated, concerns have increased with the risk of severe toxicities of these TKIs. Indeed, severe toxicities associated with TKIs, such as ATEs, VTEs and fatal adverse events, have been reported in clinical trials [15]. And several previously published meta-analyses had demonstrated that TKIs treatment increased the risk of developing ATEs [16, 17], VTEs [18–20] and FAEs [21, 22] in cancer patients. However, to our best knowledge, the overall incidence and risk of ATEs and VETs associated with TKIs in advanced/metastatic TCs patients remains undetermined. As a result, we conduct the present meta-analysis to investigate the overall incidence and risk of ATEs and VTEs in these patients receiving TKIs.

## MATERIALS AND METHODS

### Data source

Following the Preferred Reporting Items for Systematic Reviews and Meta-Analysis (PRISMA) statement [23], we performed an independent review of related citations from Pubmed, Embase, and Cochrane Library electronic databases up to August 2017. Search strategy used the following limits alone, or in combination: 1 terms describing cancer (“cancer”, “carcinoma”, and “neoplasm”); 2 terms describing thyroid tumor (“thyroid tumor”, “thyroid neoplasm”); 3 tyrosine kinase inhibitors (ie, “sorafenib”, “vandetanib”, “pazopanib”, “cediranib”, “sunitinib”, “axitinib”, “regorafenib”); 4 therapy line (ie, “first-line”, “previously treated”, “refractory”, “second-line”); 5 clinical trials (ie, “prospective”, “clinical”, “human”, or “random”). The search was restricted to clinical trials published in English. Additionally relevant articles in the reference lists of recent meta-analyses that investigated TKIs in TCs patients were also searched. In order to avoid duplication, only the most complete, recent was considered for analysis.

### Clinical endpoints definition

The following adverse outcomes were considered as VTEs/ATEs and included in the main analysis: thrombosis/thrombus/embolism (excluded vascular access related-thrombosis if reported separately), arterial thrombosis, cerebral infarct, cerebral ischemia, cerebrovascular accident, myocardial infarction and myocardial ischemia. Trials were considered for high grade (grade ≥ 3) ATE/VTEs based on Common Terminology Criteria for Adverse Events (CTCAE) v. 2.0 or 3.0. Trials that either did not list ATEs/VTEs as an adverse event or reported no ATEs/VTEs in all arms were excluded.

### Study selection

The primary objective of present study was to evaluate the overall incidence of ATEs and VTEs associated with TKIs, and the association between TKIs treatment and ATEs/VTEs in advanced TCs patients; The trials that met the following criteria were chosen for the analysis: prospective (phase II and III) clinical trials in TCs patients, patients assigned to treatment with TKIs alone, and safety data available for VTEs and ATEs. Phase I studies were excluded because of the different drug dosage and the relatively small number of patients enrolled in the trials, in line with several other meta-analyses carried out in this context [19, 20, 24].

### Data extraction

Data abstraction was conducted independently by two investigators, and any discrepancy between the reviewers was resolved by consensus. The numbers of ATEs/VTEs in both treatment and control arms were extracted from text or appendix of the trial publications. For multiple reports of the same trial, we used data from the longest follow-up. For all eligible trials, we also extracted the following information: first author’s name, year of publication, trial phase, number of enrolled subjects, treatment arms, number of patients in treatment and controlled groups, median age, and median progression-free survival.

### Statistical method

The primary summary measures were incidence, Peto odds ratios (ORs), and corresponding 95% CIs. All statistical analyses were performed by using Version 2 of the Comprehensive MetaAnalysis program (Biostat, Englewood, NJ).For the calculation of incidence, the number of patients with ATEs/VTEs in TKIs group and the total number of patients receiving TKIs were extracted; the proportion of patients with ATEs/VTEs and 95% confidence interval (CI) were derived for each study. To calculate Peto ORs, patients assigned to TKIs were compared only with those assigned to placebo treatment in the same trial. We used the Peto method to calculate odds ratio (ORs) and 95% CI confidence intervals (CIs) because this method was less biased and more powerful than other methods for rare events [25]. Between-study heterogeneity was estimated using the χ^2^-based *Q* statistic [26]. Statistical heterogeneity between trials was assessed by the *I*^2^ statistic method. A statistical test with a *p*-value less than 0.05 was considered significant. Study quality was assessed by using the Jadadscale based on the reporting of the studies’ methods and results [27].

## RESULTS

### Search results

**Figure 1:**
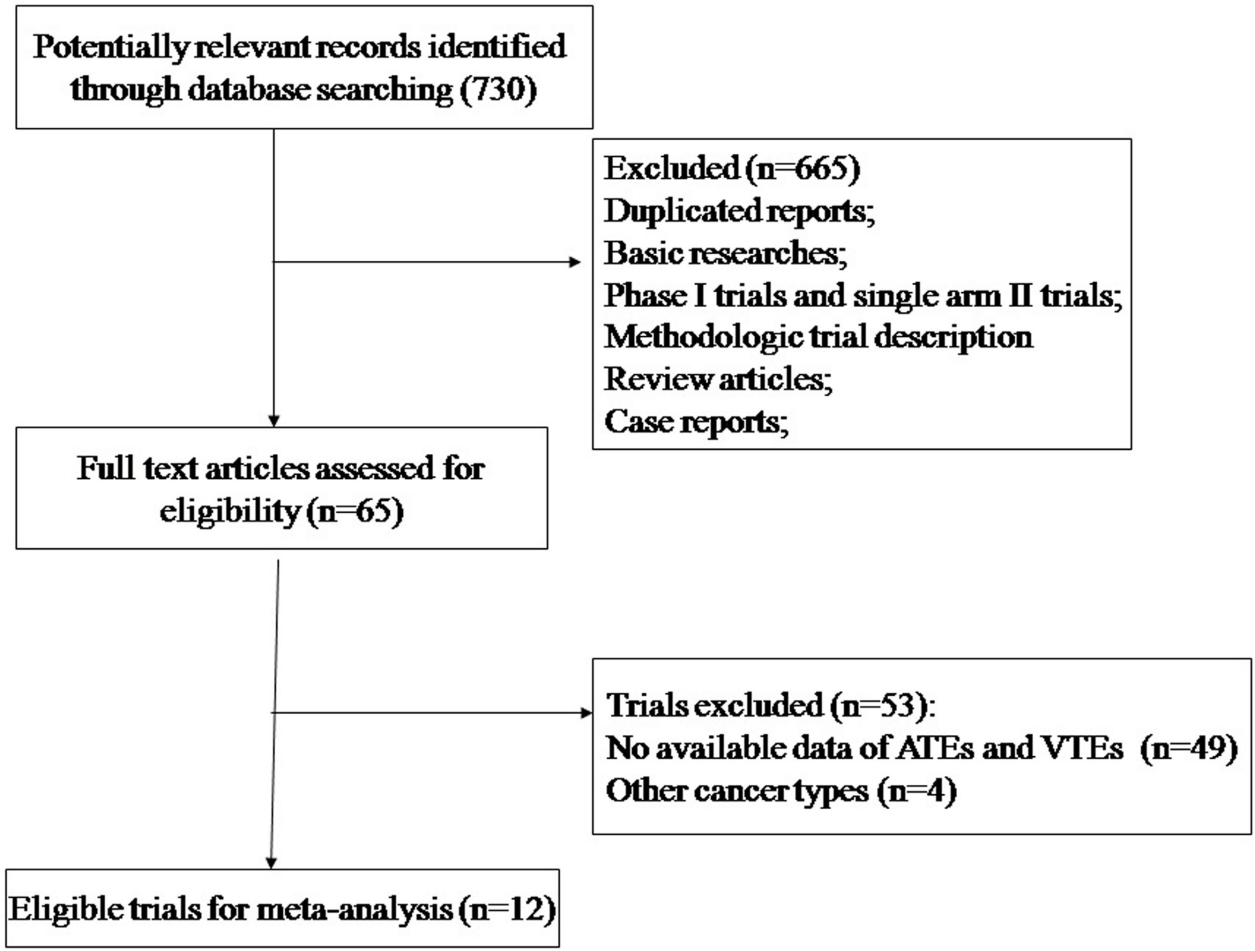
Studies eligible for inclusion in the meta-analysis.

Our search yielded a total of 192 potentially relevant studies with TKIs. Initially, 179 trials were excluded for at least one of the following reasons: absence of data on VTEs and ATEs, duplicate trials, phase I trials, nonrandomized trials, review articles, observational studies, case reports, editorials, letters and commentaries. Finally, 12 trials were considered highly relevant for the meta-analysis (phase II and III trials reporting VTEs and ATEs in the toxicity section of the publication) [28–39]. Figure 1 showed the flow diagram of trial’s selection progress according to PRISMA guidelines.

The baseline characteristics of each trial were presented in Table 1. Drug dosage and schedule were those currently approved (sorafenib 400 mg bid po; vandetanib 300 mg bid po; lenvatinib 24 mg qd po; cabozantinib 140 mg qd po). A total of 1781 patients were available for the meta-analysis; 1232 of these patients were assigned to treatment arms and 549 to placebo arms. The quality of the four randomized controlled trials was high. All of these trials were double-blinded, placebo-controlled trials, thus had a Jadad score of 5.

**Table 1:** Baseline characteristics of 12 included trials

**authors**	**phase**	**total**	**treatment arms**	**median age (years)**	**median PFS**	**No. for analysis**
**Lam E.T. et al. 2010 [40]**	II	16	sorafenib 400 mg bid po	60	17.9	16
**Wells Jr S.A. et al. 2012 [39]**	III	331	vandetanib 300 mg qd po	50.7	30.5	231
			placebo	53.4	19.3	100
**Savvides P. et al. 2013 [37]**	II		sorafenib 400 mg bid po	59	1.9	20
**Elisei R. et al. 2013 [38]**	III	330	cabozantinib 140 mg qd po	55	11.4	214
			placebo	55	4	109
**Brose M.S. et al. 2014 [36]**	III	416	sorafenib 400 mg bid po	63	10.8	207
			placebo	63	5.8	209
**Cohen E.E.W. et al. 2014 [35]**	II	60	axitnib 5 mg bid po	59	15	60
**Cabanillas M.E. et al. 2015 [30]**	II	58	lenvatinib 24 mg qd po	63	12.6	58
**Schlumberger M. et al. 2015 [31]**	III	392	lenvatinib 24 mg qd po	64	18.3	261
			placebo	61	3.6	131
**Bikas A. et al. 2016 [32]**	II	23	sunitnib 50 mg qd	61	8	23
**Schlumberger M. et al. 2016 [33]**	II	59	lenvatinib 24 mg qd po	51.6	9	59
**Cabonillas M.E. et al. 2017 [34]**	II	25	cabozantinib 140 mg qd po	64	12.7	25
**Ravaud A. et al. 2017 [29]**	II	71	sunitinib 50 mg qd	66	13.1	71

Abbreviation: PFS, progression-free survival.

### Incidence of high-grade ATEs and VTEs

**Figure 2:**
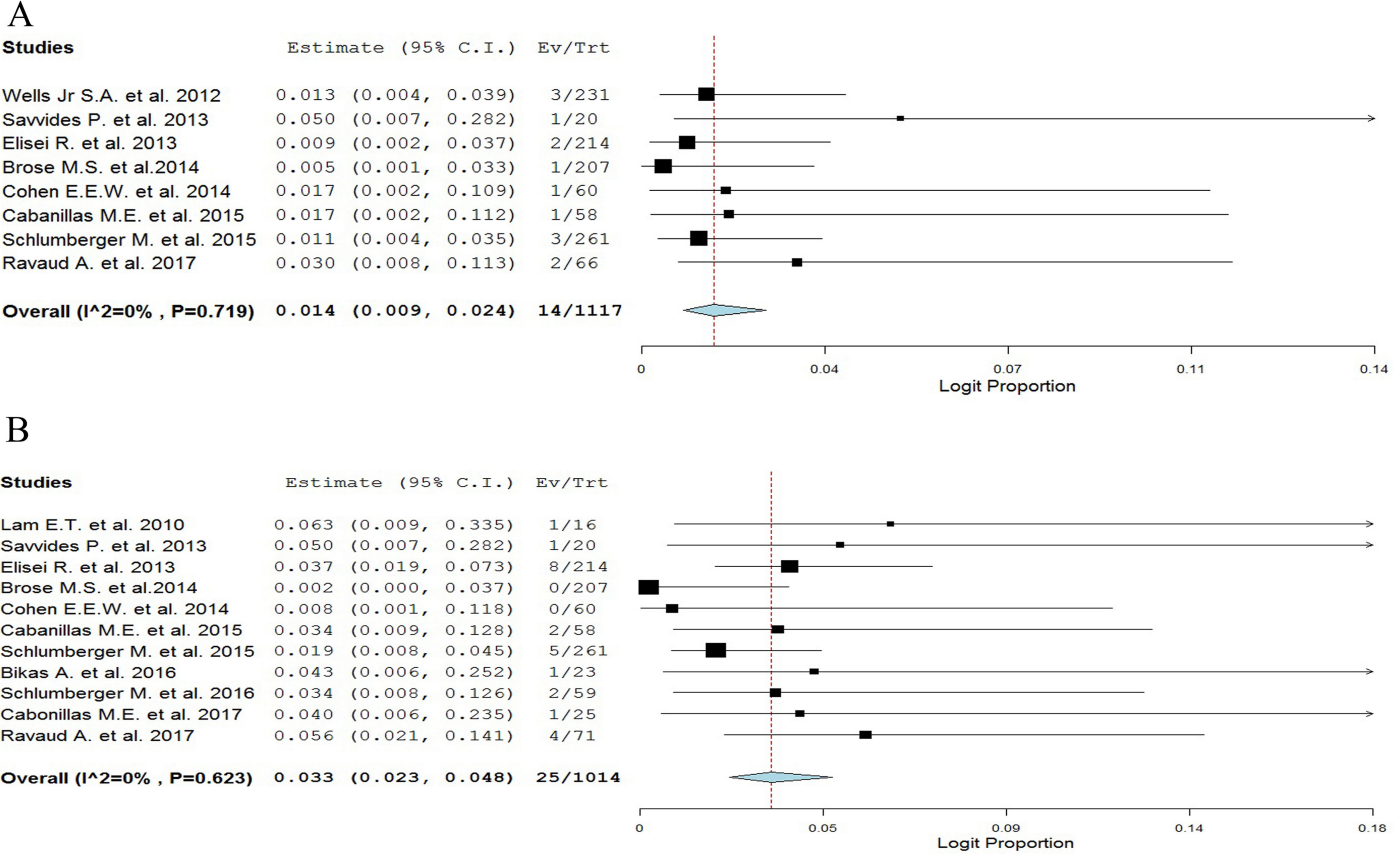
Overall incidences of ATEs and VTEs associated with TKIs in TCs patients.

A total of 1,117 patients (eight trials) were considered for the incidence analysis of high-grade ATEs. Severe ATEs occurred in 14 of 1117 patients, showing an incidence of 1.4% (95% CI: 0.9–2.4%, Figure 2A).

A total of 1,014 patients (eleven trials) were considered for the incidence analysis of high-grade VTEs. Severe VTEs occurred in 25 of 1,014 patients, showing an incidence of 3.3% (95% CI: 2.3–4.8%, Figure 2B).

### Relative risk of high-grade ATEs and VTEs

**Figure 3:**
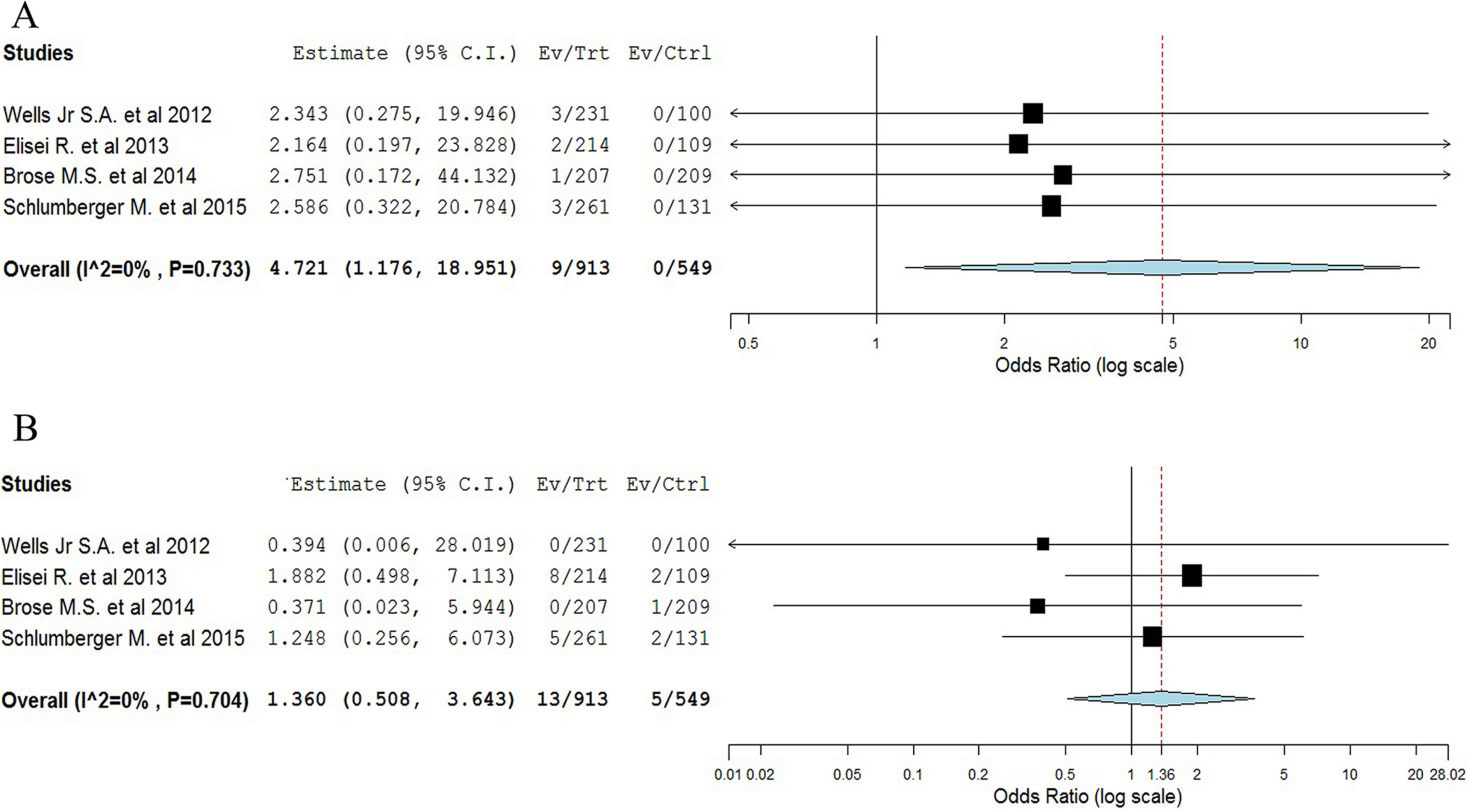
Meta-analysis of ATEs/VTEs associated with TKIs versus placebo in TCs patients.

Four randomized studies were available to calculate the Peto OR of high-grade ATEs in patients assigned to TKIs versus placebo in the same trial. The meta-analysis showed that the summary Peto OR of high-grade ATEs in TKIs versus placebo arms was 4.72 (95% CI 1.18–18.95; *P* = 0.029, Figure 3A), suggesting a nearly 5 times higher risk of developing high-grade ATEs with TKIs compared with placebo. The test for heterogeneity was not significant (I^2^ = 0%, *p* = 0.73).

Four randomized trials were available to calculate the Peto OR of high-grade VTEs. The meta-analysis showed that the summary Peto OR of VTEs in experimental versus control arms was a non-significant1.36 (95% CI 0.51–3.64; *P* = 0.54, Figure 3B). The test for heterogeneity was not significant (I^2^ = 0%, *P* = 0.70).

### Specific cause of ATEs and VTEs

Individual specified and non-specified causes of ATEs/VTEs events were listed in Table 2. A total of 24 severe VTEs occurred on the treatment arms and 5 on the controlled arms, and the most common causes of VTEs associated with TKIs were venous thrombosis (58.3%) and pulmonary embolus (33.3%). For high-grade ATEs, a total of 14 high-grade ATEs occurred on the TKIs arms, and there was none incidence of ATEs in the placebo arms. The most common of specific ATEs was myocardial infarction (28.6%) and ischemic cerebrovascular events (21.4%).

**Table 2:** ATEs and VTEs by specific types

	**Events on the TKIs arms**	**Events on placebo arms**
Pulmonary embolus	8	3
Thrombosis	2	0
Venous thrombosis	14	2
Arterial thrombosis	2	0
Cardiac ischemia	1	0
Ischemic cerebrovascular events	3	0
Myocardial infarction	4	0
Cerebrovascular accident	1	0
Cerebral ischemia	1	0
Ischemic stroke	1	0
Transient ischemic attack	1	0

## DISCUSSION

The treatment of RAI-refractory TCs tends to be very challenging due to limited treatment options [40]. Recently, different kinds of novel TKIs have been investigated in cancer patients and shown improving overall survival and progression-free survival. Indeed, two previously published meta-analyses had demonstrated that TKIs treatment in advanced/metastatic thyroid cancer patients was associated with a significantly improved overall survival and progression-free survival, and higher incidence of diarrhea, rash, and hypertension was observed in TKIs treatment group [41, 42]. But the overall incidence and risk of severe ATEs/VTEs associated with TKIs in TCs patients remains undetermined, although these toxicities have been observed in clinical practice.

To our best knowledge, our study is the first comprehensive meta-analysis to evaluate the VTEs and ATEs in advanced TCs patients receiving TKIs. Our analysis of data from 12 trials, including four randomized controlled trials and eight phase II single arm trials, demonstrates that overall incidence of high-grade ATEs and VTEs associated with TKIs are 1.4% and 3.3%, respectively. Previous meta-analysis consistently supports a small increase in ATEs from TKIs across a range of advanced solid tumors. The RR for ATEs with sunitinib or sorafenib was 3.03 in one trial level meta-analysis (*p* = 0.015) [16]. In consistent with previous results, our study shows that TKIs treatment in advanced or metastatic thyroid cancer significantly increases the risk of developing high-grade ATEs (Peto OR 4.72, *p* = 0.029). Conversely, data do not consistently support an elevation in risk of VTEs with TKIs in comparison with placebo [18, 19]. In a meta-analysis (*n* = 4,430) studied vascular endothelial growth factor receptor-TKI and reported an overall incidence of VTE of 3% and RR = 0.91, *p* = 0.64) in solid tumors. Similarly, the present study demonstrates that TKIs treatment in advanced or metastatic thyroid cancer does not significantly increases the risk of developing high-grade VTEs in comparison with placebo (Peto OR 1.36, *p* = 0.54). Although the absolute incidence rate of ATEs is very low, the potential long-term survival benefit of TKIs might outweigh the risk. As a result, it is important that healthcare practitioners and patients recognize the risk of ATEs and maximize the clinical benefits of TKIs in TCs patients. We then investigate the specific causes of ATEs associated with TKIs, and find that most common of specific ATEs is myocardial infarction (28.6%) and ischemic cerebrovascular events (21.4%), respectively. On the basis of our findings, clinicians should fully treat patients with active cardiac or cerebrovascular disease to reduce the risk of severe ATEs before the initiation of TKIs in TCs patients, and must monitor patients during the course of TKIs treatment.

The present study has several limitations. Firstly, our study is a meta-analysis of published data, thus individual patient information is not available. Second, toxicities are not the primary end-point in all included trials, and the process of attribution of ATEs/VTEs’ causality might be a potential source of bias. However, all of the included RCTs are double blinded and placebo controlled trials, which have low risk of bias. Finally, TCs patients receiving different kinds of TKIs are included for analysis, which would increase the clinical heterogeneity among included trials. However, it also makes the interpretation of a meta-analysis more problematic.

In conclusion, this study demonstrates that TKIs treatment in advanced TCs patients is associated with a significant increase of high-grade ATEs, but not for VTEs. Given the increasing use of TKIs in TCs patients, it is important for physicians and patients to be aware of the risk of ATEs and prevent accordingly, especially those caused by cardiac toxicity, to maximize the clinical benefits of TKIs in these patients.
